# Alternative neural systems: What is a neuron? (Ctenophores, sponges and placozoans)

**DOI:** 10.3389/fcell.2022.1071961

**Published:** 2022-12-23

**Authors:** Leonid L. Moroz, Daria Y. Romanova

**Affiliations:** ^1^ Departments of Neuroscience and McKnight Brain Institute, University of Florida, Gainesville, FL, United States; ^2^ Whitney Laboratory for Marine Bioscience, University of Florida, St. Augustine, FL, United States; ^3^ Institute of Higher Nervous Activity and Neurophysiology of RAS, 5A Butlerova, Moscow, Russia

**Keywords:** ctenophora, placozoa, porifera, nervous system evolution, synapse, innexins, neurotransmitters, homology

## Abstract

How to make a neuron, a synapse, and a neural circuit? Is there only one ‘design’ for a neural architecture with a universally shared genomic blueprint across species? The brief answer is “No.” Four early divergent lineages from the nerveless common ancestor of all animals independently evolved distinct neuroid-type integrative systems. One of these is a subset of neural nets in comb jellies with unique synapses; the second lineage is the well-known Cnidaria + Bilateria; the two others are non-synaptic neuroid systems in sponges and placozoans. By integrating scRNA-seq and microscopy data, we revise the definition of neurons as synaptically-coupled polarized and highly heterogenous secretory cells at the top of behavioral hierarchies with learning capabilities. This physiological (not phylogenetic) definition separates ‘true’ neurons from non-synaptically and gap junction-coupled integrative systems executing more stereotyped behaviors. Growing evidence supports the hypothesis of multiple origins of neurons and synapses. Thus, many non-bilaterian and bilaterian neuronal classes, circuits or systems are considered functional rather than genetic categories, composed of non-homologous cell types. In summary, little-explored examples of convergent neuronal evolution in representatives of early branching metazoans provide conceptually novel microanatomical and physiological architectures of behavioral controls in animals with prospects of neuro-engineering and synthetic biology.

## Introduction

“The current definition of a nervous system has negative consequences in the field of evolutionary biology that preclude discussing the processes of convergent evolution in multicellular organisms. A phylogenetic definition of an organism’s biological system prevents us from considering whether that system has emerged in other organisms outside that definition.”- Miguel-Tome and Llinas (2021).

The origins and rise of neuronal complexity are among the rarest yet globally impactful life transitions, and these events likely occurred over 570–530 million years ago near the Cambrian boundary. Despite more than a century of comparative research, the mechanisms and pathways of nervous system evoluton among 30 + animal phyla are elusive (Moroz, 2018). From broad genomic and comparative viewpoints, we still do not have an agreed definition of a neuron. As a result, understanding astonishing neuronal diversity is a critical experimental endeavor and theoretical challenge by itself.

All studied extant neural systems contain highly heterogeneous neuronal populations with multiple cell types, which is the hallmark of any neural organization. Every neuron in a given nervous system can be unique regarding its connectivity, functions, morphology, and gene expression patterns. But, the rules underlying the neuronal heterogeneity and the entire scope of the neuronal diversity across phyla remain unknown, calling for novel unbiassed NeuroSystematics and/or Periodic System of Neurons with predictive power (Moroz, 2018).

*How* different are neurons, and more importantly, *why* are they so different? The evolutionary hypothesis can be as follows. Neurons are different not only because they have different functions but also because neurons, as evolutionary units (Arendt, 2008; Arendt et al., 2016), have different genealogies with distinct gene regulatory programs and signal molecules (neurotransmitters) reflecting their parallel evolution at the broadest evolutionary scale (Moroz, 2009). Studied by scRNA-seq vertebrate [e.g., (Raj et al., 2018; Armand et al., 2021; Bandler et al., 2022; Delgado et al., 2022)] and invertebrate neuronal cell types [e.g., (Corrales et al., 2022; Dillon et al., 2022)] are organized in hierarchical trees but with unknown principles and uncertain criteria for homologization across phyla. How this diversity evolved in the first place is also unknown because strategies to probe ancestral neuronal specification events are limited.

## Pre-bilaterian metazoans as essential reference species for fundamental neuroscience

**FIGURE 1:**
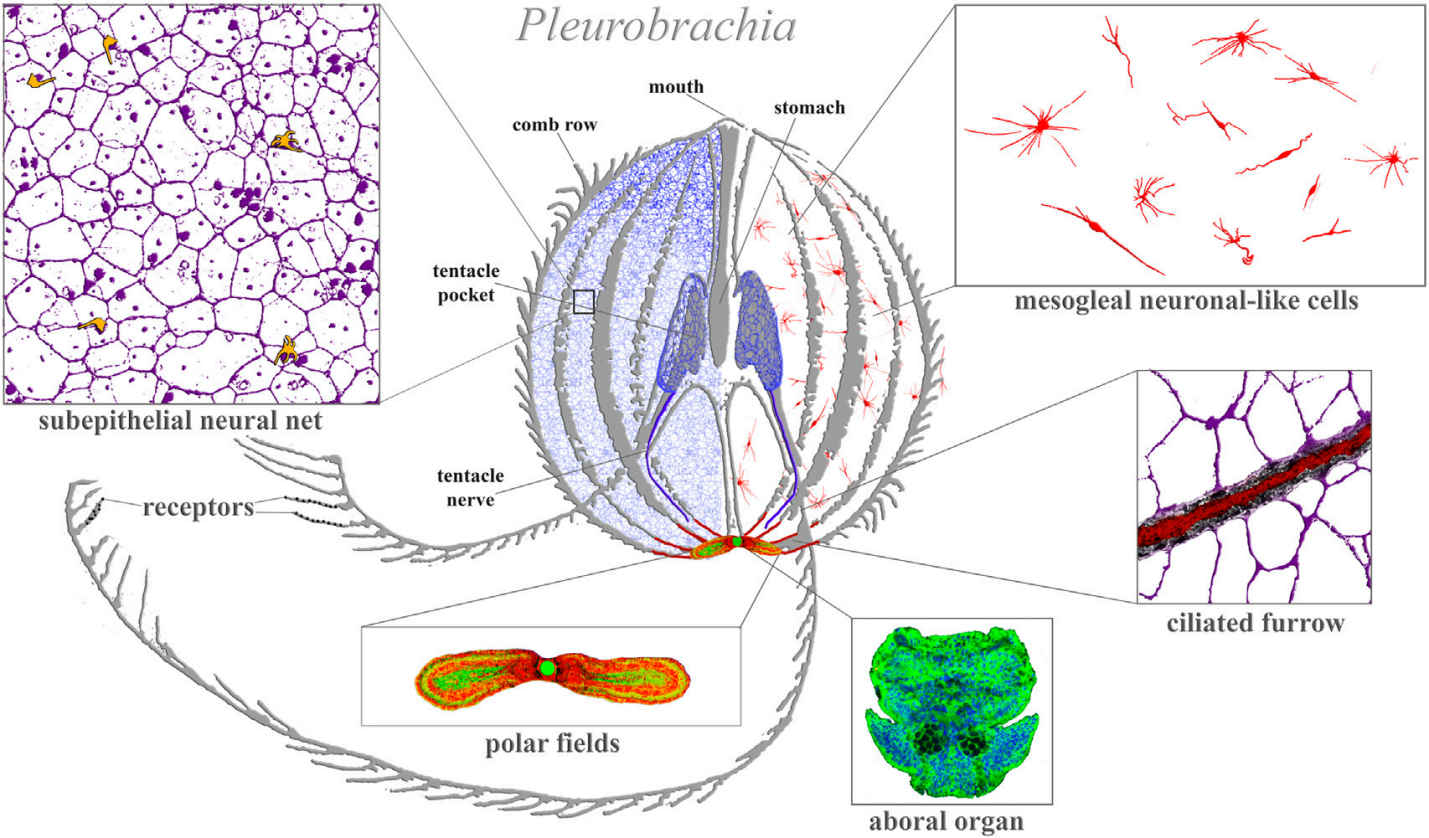
Ctenophore neural systems. As an illustrated example, the schematic diagram is based on the recent study of the cydippid *Pleurobrachia bachei* (Norekian and Moroz, 2016; 2019b). Different colors indicate different cellular populations. Most neurons and receptors (yellow) are located within the subepithelial neural net in the skin (blue, magenta) and tentacle shields with two tentacular nerves (dark blue). There are two concentrations of neural elements: one in the aboral organ (green) with densely packed neurons and other cell types (the elementary brain?); and the second in the polar fields putative chemosensory structures (yellow/green, red marks phalloidin-labeled elements). The mesoglea has a diffuse population of neuron-like cells (red). Eight ciliated furrows (conductive ciliated cells—red lines) connect the aboral organ with comb plates. The ciliated furrows are closely associated with neural net elements (insert) and are possible under neuronal control.

**FIGURE 2:**
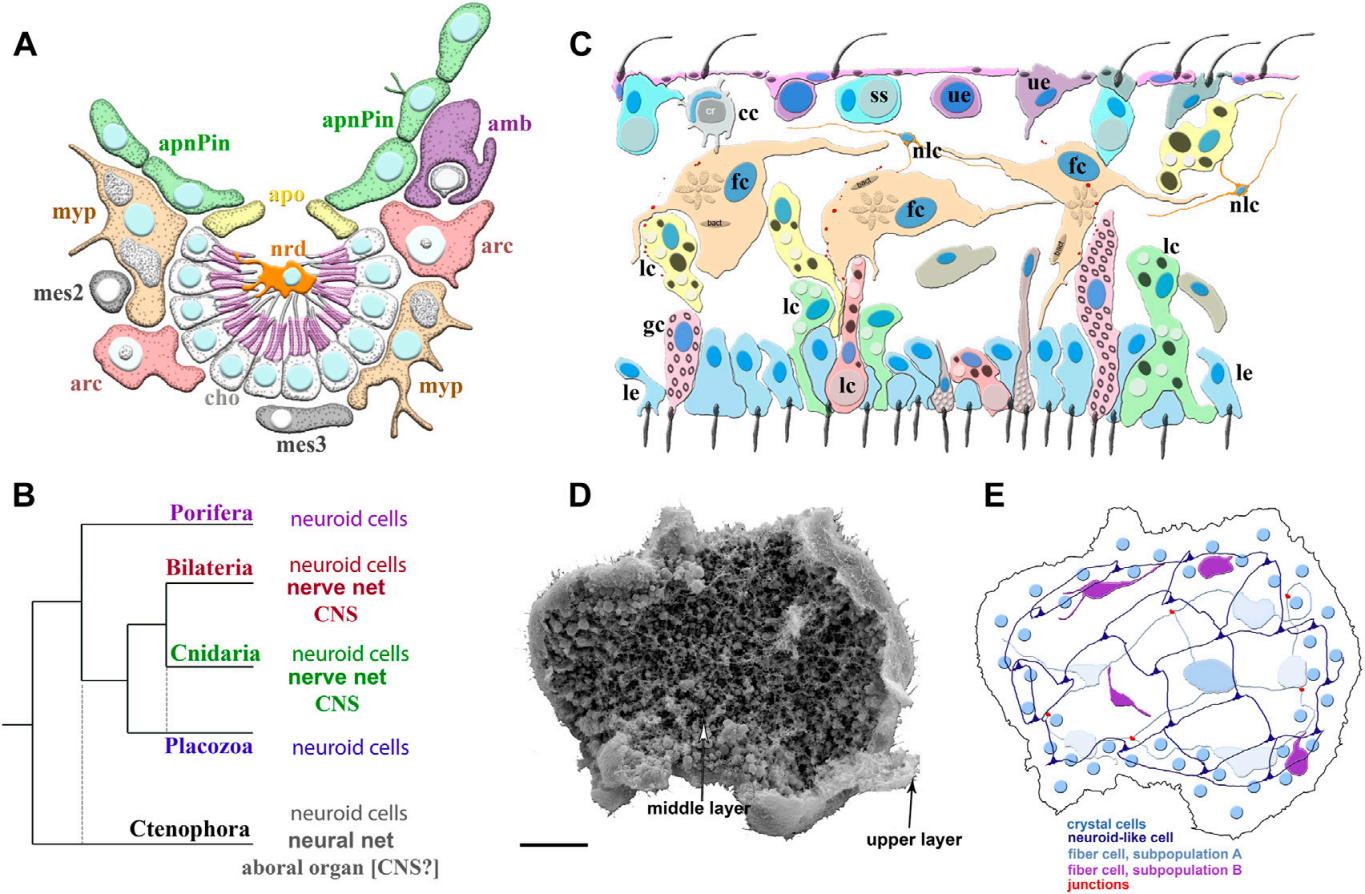
Poriferan and Placozoan neuroid systems. **(A)** Different cell types (different colors) were identified using scRNA-seq in the demosponge *Spongilla lacustris* (Musser et al., 2021): apnPin—apendopinacocytes; apo—apopylar cells; amb—amoebocytes; arc—archeocytes; cho—choanocytes; mes2 and mes3—mesocytes; myp—myopeptidocytes; nrd—neuroid cells (orange). The neuroid cells are located in the center of the choanocyte chamber, make connections to choanocytes, and might be involved in their control as neuronal-like elements. These neuroid cells contain secretory apparatus and vesicles. However, the transcriptome profiles of these neuroid cells are remarkably different subset from other known neural/neuroid-type cells in metazoans (Nakanishi et al., 2015; Musser et al., 2021), suggesting that these are sponge-specific innovations with no apparent homologs in other animals. The nature of these cellular interactions is unknown. **(B)** The emerging diversity of cell types in the placozoans. The diagram is based on recent ultrastructural studies (Smith et al., 2014; Smith et al., 2015; Mayorova et al., 2018; Mayorova et al., 2019; Romanova et al., 2021). Several morphologically distinct cell types were identified: cc—crystal cells; fc—fiber cells; gc—gland cells; lc—lipophil cells; le—lower epithelial cells; nlc-neuroid-like cells, which were previously labeled as stellate-like cells (Romanova et al., 2021); ss—shiny spheres; ue—upper epithelial cells. **(C1)** Scanning electron microscopy of *Trichoplax*—an animal without an upper cell layer. The photo shows the spatial organization of a complex meshwork formed by elements above the middle layer and the upper layers of the animal. Distributed net-like structures were formed by processes of different subtypes of fiber cells and stellate-like cells, which we also named neuroid-like cells. Some heterogeneity of fiber and neuroid-like cells is anticipated from recent ultrastructural studies (Romanova et al., 2021). **(C2)** Schematics of the spatial distribution of different subtypes of fiber, neuroid-like cells, and their processes. All these cells might form in a net-like structure above the upper layer with crystal cells as a distributed integrative system. This reconstruction is based on (Romanova et al., 2021; Romanova et al., 2022) and unpublished data. Scale: 20 μm.

Here, we must stress the need to study *reference* species (vs. ‘*model*’ organisms) as taxonomically diverse evolutionary groups with a wide-ranging spectrum of ecological adaptations and novelties in neural architecture (Striedter et al., 2014). Representatives of three early branching metazoans lineages, placozoans (the phylum Placozoa), sponges (Porifera), and comb jellies (Ctenophora) are the most critical reference species to reconstruct the origins of animal innovations, which led to the formation of neural systems (Figure 1 and Figure 2). Regrettably, these pre-bilaterians belong to the most enigmatic animals in neuroscience; they are often viewed as less relevant for biomedical questions with noticeable underfunding.

The position of comb jellies as the sister lineage to all Metazoa (Figure 2B) has been supported by independent large-scale phylogenomic studies: this is the Ctenophora-first hypothesis (Whelan et al., 2015; Halanych et al., 2016; Whelan et al., 2017; Laumer et al., 2019; Fernandez and Gabaldon, 2020; Li et al., 2021). Other evolution models challenge this topology of the animal tree of life and place nerveless sponges as the earliest diverged lineage (Sponge-first hypothesis), followed by ctenophores with developed neural systems, and then again nerveless placozoans (Telford et al., 2016; Kapli and Telford, 2020; Redmond and McLysaght, 2021; Giacomelli et al., 2022). Morphological and hydrodynamic views of animal evolution also emphasize the sponge-first hypothesis (Nielsen, 2019, 2022). However, “Systematic and standardized testing of diverse phylogenetic models suggests that we should be skeptical of Porifera-sister results both because they are recovered under such narrow conditions and because the models in these conditions fit the data no better than other models that recover Ctenophora-sister” (Li et al., 2021).

Regardless of these two conflicting phylogenetic hypotheses, the unique architecture of extant neural systems in ctenophores strongly supports the hypothesis of independent origins of neurons and synapses over 550 million years of animal evolution (Moroz, 2014b; Moroz et al., 2014; Moroz, 2015; Moroz and Kohn, 2016). According to this scenario, a nerveless organism was the last common ancestor of all extant animals (LCAA or the urmetazoan). Ancestors of ctenophores evolved a distinct neuronal organization to control complex ciliated locomotion (by multiple comb plates) and other behaviors in these predatory animals. Sponges and placozoans *remained* nerveless by occupying different ecological niches (Nielsen, 2022; Romanova et al., 2022), with unique adaptations based on orchestrating cilia beating and expanding non-muscular contractivity (Leys, 2015; Smith et al., 2015; Armon et al., 2018; Leys et al., 2019; Kornder et al., 2022; Nielsen, 2022). The common ancestors of cnidarians and bilaterians also evolved neural cell types to integrate the operation of multiple muscular, ciliated, and secretory effectors as adaptations that might accompany the increased body sizes of early animals and complex movements. Nevertheless, *four* early divergent lineages from the LCAA *independently* evolved alternative neuroid-type integrative systems (as summarized in Figure 1 and Figure 2).

This hypothesis implies dissimilar gene regulatory programs with unique combinations of transcription factors and other regulators controlling terminal specifications of neurons in ctenophores, cnidarians and bilaterians, respectively, as well as parallel recruitment of neurotransmitters and other signal molecules. For example, serotonin, dopamine, noradrenaline, octopamine, histamine, and acetylcholine act as neurotransmitters in bilaterians but not in ctenophores or cnidarians (Moroz et al., 2014; Moroz et al., 2021b). None of the ctenophore (neuro) peptide homologs are found in any other animal phylum (Moroz et al., 2014; Moroz and Kohn, 2016; Sachkova et al., 2021; Hayakawa et al., 2022). Furthermore, known bilaterian neuronal markers and many relevant transcription factors either absent in ctenophores or if present were not expressed in neurons (Moroz et al., 2014; Moroz and Kohn, 2015). This line of evidence can be further experimentally tested to falsify or support the hypothesis of the independent origins of neurons. The initial functional genomic/transcriptomic analyses of sequenced from 30 + species (Whelan et al., 2017) revealed distinct molecular toolkits associated with ctenophores’ neuromuscular and synaptic organizations (Moroz et al., 2014; Moroz and Kohn, 2016; Moroz et al., 2020b).

Several authors argue for a single origin of neurons and subsequent loss of neuronal and muscle cell types in placozoans and sponges (Rokas, 2013; Ryan, 2014; Ryan and Chiodin, 2015), irrespective of any modern phylogeny. The functional reasons for such ‘neuronal’ losses in free-living (non-parasitic) animals are unclear, and in our opinion, this hypothesis lacks sufficient rationale and support. A few selected genes involved in the excitability, secretion, or reception of some eukaryotic signal molecules cannot be used for the homologization of neural structures across metazoans. Moreover, the absence of pan-neuronal gene-/molecular markers (Moroz and Kohn, 2015) and shared gene regulatory programs leading to neuronal specification favor independent origins of neurons in ctenophores and the common ancestor of cnidarians + bilaterians (Moroz et al., 2014; Moroz and Kohn, 2016). Because cnidarians have both endoderm- and ectoderm-derived neuronal populations, there is also a possibility of cnidarian-specific neuronal cell types (Arendt, 2019).

Predictably, one or few neuronal cell-type lineages could be more evolutionarily ancient than others, but comparative data are lacking. Therefore, all hypotheses and evolutionary scenarios outlined above should be rigorously tested by the broadest sampling and molecular/physiological characterizations of all cell types across major taxonomical groups of extant animals. Ideally, this goal should include analysis of all about 100 animal classes and even orders, with extensive scRNA-seq profiling and cell-type homology testing, focusing on non-bilaterian metazoans as a start. This monumental task requires decades of research. The actual outcomes will lead to the unbiased phylogenomic classification of neuronal and other cell types across metazoans (=NeuroSystematics). NeuroSystematic would be the foundation to unravel genomic bases controlling neuronal identity and neuronal circuit evolution with the predictive power of novel cell phenotypes—a hypothetical Periodic System of Neurons (Moroz, 2018). Promising approaches include scRNA-seq, tools of statistical geometry (Liang et al., 2015) and novel algorithms (Tarashansky et al., 2021) to find conservative Character Identity Networks (Wagner, 2007; Wagner, 2014) defining homologous cell types (Musser et al., 2021; Li et al., 2022); eventually targeting reconstructions of neuronal ancestry (Moroz, 2009; 2014a; Arendt et al., 2015).

Admittedly, the cellular and molecular bases of behaviors in sponges, placozoans, and ctenophores are so remarkably distinct compared to the rest of the animals that it would be advantageous to explore the concept of 3 separate ‘designs’ for neuroid architectures (Figure 1 and Figure 2) that evolved in parallel from the late Precambrian time.

Indeed, placozoans show remarkable complex and highly integrated behaviors of numerous cellular populations without any recognizable synaptic organization or gap junctions, implying highly coordinated paracrine secretion and volume transmission. For example, during *Trichoplax* feeding, hundreds of cells and cilia reversible change their behaviors, and some of these cells could also be chemosensory such as gland cells in the rim (Smith et al., 2015); perhaps co-acting together with a meshwork of fiber and other neuroid cells and forming one type of the alternative integrative system (Figures 2C–E).

Relatively complex behaviors present in the demosponge (*Amphimedon*) larvae eventually leading to settlement and metamorphosis. Sensing environmental cues can be mediated by specialized epithelial secretory flask cells, which possess a cilium and F-actin-rich protrusion, secretory vesicles, and neurite-like processes (Nakanishi et al., 2015). These cells share similar structural features with sensory-neurosecretory cells in cnidarian and bilaterian larvae, implying the hypothesis of their shared ancestry with eumetazoans (Nakanishi et al., 2015). It is intriguing to view these flask-like cells as evolutionary predecessors of some neuronal types or, more likely, analogs of such predecessors. Early diverged ancestral animal lineages might share some homologous molecular components of the sensory and secretory machinery. On the other hand, the equally feasible scenario can be co-options of functionally similar molecular complexes for similar chemoreceptive tasks forming a case for convergent evolution based on the modular organization of eukaryotic signaling systems (Arendt, 2020).

After the metamorphosis, the larval flask cells can be transdifferentiated into stem-like archeocytes and, subsequentially to other cell types such as choanocytes and others (Nakanishi et al., 2014). Interestingly, recently discovered, by scRNA-seq, adult neuroid cells in *Spongilla* (Figure 2A) also belong to the broad archeocyte/amoebocyte family (Musser et al., 2021). Although these correlations might hint at the common ancestry of both types of neuroid cells in two species of sponges, we need reliable evidence for their homologization using future molecular/scRNA-seq data.

Similarly, there are no reliable molecular markers for placozoan fiber or other neuroid-like cells (Figures 2C,E). Moreover, sponge flask cells and placozoan fiber/gland cells likely utilize species-/lineage-specific secretory molecules, absent in ctenophores, cnidarians or bilaterians (Srivastava et al., 2008; Srivastava et al., 2010; Moroz et al., 2014; Nakanishi et al., 2015; Smith et al., 2015; Musser et al., 2021). As a result, for these two groups of nerveless animals, we use the term alternative integrative systems, considering the hypothesis of their parallel evolution. At the same time, the control of metamorphosis in ancestral larvae by different types of sensory-secretory cells or conceptually similar control of feeding and digestion in early animals could be universal exaptations underlying the origins of true neural signaling and nervous systems.

Equally important to this goal would be revisiting the terminology and definition of neurons, nervous systems, and synapses. There are two options in this endeavor. The first is broadening the definition of neural systems to include plants (Baluska, 2009; Baluska and Mancuso, 2009b; a;c; Baluska et al., 2009; Baluska, 2010; Baluska et al., 2010; Baluska and Mancuso, 2021) and, perhaps, other eukaryotes, as was recently proposed (Miguel-Tome and Llinas, 2021). The second option is narrowing the physiological definition of neural systems to animals only, but with the concept of non-homologous (to bilaterians) neuronal cell type lineages and extensive convergent evolution. More generally, the entire spectrum of alternative integrative systems in organisms should include (a) ‘true’ neuronal systems across Metazoa, considering examples of their convergent evolution as in Ctenophora (Figure 1) or distinct set of synaptic ensembles in Cnidaria (Anderson, 1985; Anderson and Grunert, 1988; Anderson and Spencer, 1989); (b) different neuroid-type conductive, (chemo) sensory and secretory cells in non-bilaterian animals (Mackie, 1970; Anderson, 1980; Anderson and Schwab, 1982; Mackie, 1990; Tamm, 2014a; Nakanishi et al., 2015; Smith et al., 2015; Moroz et al., 2021b; Musser et al., 2021; Romanova et al., 2021), Figure 2; and (c) physically or chemical coupled cell populations in non-animal groups (Miguel-Tome and Llinas, 2021).

This manuscript explores a broader definition of neurons as functional rather than genetic traits using examples of alternative neural/integrative systems in basal metazoan lineages. Here, we are paying more attention to the nervous systems of ctenophores, as the most unique from all known of neuroid-type organizations.

## What is a neuron? Chemical synapses as the hallmark of the neuronal organization?

*Practical and conceptual challenges*. Due to specific microanatomical criteria, there are no problems recognizing neurons in vertebrates, arthropods, mollusks, or worms. However, identifying diverse neuronal cell types in basal deuterostomes (e.g., hemichordates or *Xenoturbella*) and across non-bilaterians is challenging. Researchers frequently use a location by *in situ* hybridization with various markers in comparative studies. However, most mRNAs are usually not transported to neuronal processes hiding specific cell morphology such as branched neurites, characteristics for many neurons (Puthanveettil et al., 2013). Immunohistochemistry helps at a limited scale for transmitter markers since many signal molecules also operate in non-neuronal cells. And we, *a priori*, do not know that any given transmitter candidate is a neurotransmitter, i.e., a signal molecule released from neurons for information transmission. In fact, such rigorous proof of identity is absent for most neurotransmitter candidates in ctenophores and cnidarians. The unbiased identification of neurons in developmental stages and neural nets is more complicated. Plus, there are multiple non-neuronal polarized secretory cells with numerous processes.

The lack of universal pan-neuronal markers (Moroz and Kohn, 2015) adds extra challenges for identifying neuronal types in early-branched metazoans. Physiological and functional definitions of biological systems and even individual cells are widely used in textbooks and experimental biology (secretory, digestive, immune, skeletal, contractive, respiratory, circulatory, locomotory, *etc.*), but not so often in evolutionary neuroscience. Function-based terminology does not always imply homologization and direct phylogenetic relationships among respiratory or circulatory systems, for example. In contrast, it opens to multiple convergent/independent/parallel evolution instances, including the origins and diversifications of nervous systems (Moroz, 2009; Miguel-Tome and Llinas, 2021).

It is interesting to reread the 60 years old debate of two influential thinkers in evolutionary neuroscience—how to recognize and determine neurons in ctenophores.

- “G. O. Mackie: I am interested in the two types of cells in the ciliated groove. Both appear to conduct but you call one of them nerves and you say that the other conducts in a ‘neuroid’' fashion. Where do you draw the line between nerve cells and ‘neuroid’ conducting cells?
- G. A. Horridge: The epithelial cells have grown elongated and parallel. They conduct over long distances and resemble nerve cells but happen to be ciliated. I would call this an independent origin of a nerve cell, but the whole definition of nerves is in question. As soon as you trace the origin of any category down to its simplest limits, you find that your definitions become arbitrary. If you remove stones from a heap until you have four left, is that still a heap? If you remove another and you have 3, is that a heap? When you have only two left, that is probably not a heap. Similarly, when you discover progressively more elementary nervous system or follow any structure in the animal kingdom down to its simplest limits you find that your definitions are no longer simple.”—cited from (Horridge, 1966).

Morphological and molecular data over the last decade added new layers of complexities to the organization of ‘simpler’ neural systems in ctenophores. The overall neuromuscular architecture has been characterized in 11 ctenophore species representing major phyletic lineages within this group: *Euplokamis*, *Pleurobrachia*, benthic ctenophores, lobates (*Mnemiopsis* and *Bolinopsis*), *Beroe*, *Dryodora* and even unnamed species (Norekian and Moroz, 2016; 2019a; b;^2020^; ^2021^).

Figure 1 illustrates the neural organization of the cydippid *Pleurobrachia* as an example. This species has about 10,000–15,000 neurons, which form four distinct subsystems, each with unique molecular and microanatomical organization: 1) epithelial neural net with neurons arranged in specific orthogonal fashion and their branches to tentacles; 2) compact neural-like cells in the aboral organ with the gravity sensor (Tamm, 2014b) and a putative integrative center (Tamm, 1982) (=elementary brain?); 3) distinct populations of neural-type cells in the polar fields (putative chemoreceptor structures); and 4) diffuse mesogleal neuroid-like net of cells with unknown functions. There are apparent connections (including synaptic) within four subsystems (Hernandez-Nicaise, 1991). This type of organization is well-preserved across 11 studied ctenophore species with novel *lineage-specific neuronal populations*, such as those found in the feeding lobes of lobates (*Mnemiopsis* and *Bolinopsis* (Norekian and Moroz, 2021) or independently evolved giant axonal systems and striated muscles in *Euplokamis* (Norekian and Moroz, 2020). We can now distinguish more than 20 morphologically different populations of neurons and five types of receptors in each studied species (Norekian and Moroz, 2019a; b;^2020^).

Surprisingly, at least five neurons in the early post-hatching stages of *Mnemiopsis* can form a syncitium with fused plasma membranes (Sachkova et al., 2021; Burkhardt et al., 2022). Such syncytial-type of networks are relatively rare in nature. Notable exemptions include neurons of the cephalopod stellate ganglion, where their processes are fused to form giant axons (Young, 1939), syncytial neural nets in the colonial polyp *Velella* (Mackie, 1960; Mackie et al., 1988), cell-cell fusion in leech, gastropod molluscs, nematodes, mammals (Oren-Suissa et al., 2010; Giordano-Santini et al., 2016; Giordano-Santini et al., 2020). Neurite and synaptic fusion and pruning occurred during neural development and neuroplasticity in *Drosophila* (Yu and Schuldiner, 2014) and mammals (Faust et al., 2021) and might be mechanisms of adaptations for the propagation of signals. By summarizing the Neuron Doctrine, Raymon y Cajal ‘wisely considered that “neuronal discontinuity … could sustain *some exceptions*” (Cajal, 1995; Bullock et al., 2005). Coupling cells and neurites into functional syncytia might occur with and without electrical synapses (see also below). Ctenophores present the exceptional opportunity to readdress 100 years-old reticular concepts of neuronal architectures.

Whether the syncytial organization of some ctenophore larval neurons is primarily, or secondary traits remain to be determined. Does it exist in adults or other ctenophores species? In summary, the syncytial type organization, unique tripartite synapses, unique molecular toolkits, unique expression of transcription factors, and diversity of unique, ctenophore-specific neuropeptides, plus lack of majority known low molecular weight transmitters are arguments for the hypothesis of independent origins of these neural systems, as proposed earlier (Moroz, 2009; 2014a; Moroz et al., 2014; Moroz, 2021). However, in addition to neuronal systems, the ctenophore contained several neuroid conductive systems in the ciliated furrows and some mesoglea muscle-like and star-like cells (Hernandez-Nicaise, 1968; Tamm, 1973; Tamm, 1982; Tamm, 1984; Tamm and Moss, 1985; Hernandez-Nicaise, 1991; Tamm and Tamm, 2002; Tamm, 2014a; Norekian and Moroz, 2020). These cell populations return us to the 60-year Mackie-Horridge discussion of separating neurons from other neuroid-like cells.

Establishing universal criteria to define neurons is vital in analyzing the origin and evolution of nervous systems. Are there any such universal criteria? Is there a universal molecular toolkit that makes a neuron? What is a neuron from the genomic viewpoint? Available scRNA-seq data alone did not provide practical markers to recognize neurons (Sebe-Pedros et al., 2018). Neither action potentials nor exocytosis/receptive molecular components of synapses are absolute prerequisites of neurons, as they were found in sponges and placozoans (Leys et al., 1999; Sakarya et al., 2007; Kosik, 2009; Srivastava et al., 2010; Leys, 2015; Varoqueaux and Fasshauer, 2017; Wong et al., 2019; Romanova et al., 2020). Historically, there can be many transition stages within the same evolutionary cell lineage, from a simple secretory cell without well-defined processes to a highly polarized neuron with hundreds of specialized neurites and thousands of synapses.

Regarding the definition of neurons, the combination of the following features of neurons should be considered. Still, they need to be carefully validated in a broad comparative survey that includes representatives of all basal metazoan lineages.

(1) Neurons are asymmetrical, highly polarized secretory cells, which persistently maintain one or multiple cellular processes (neurites) as well as distinct compartments specialized for directed information processing to other cell types and demonstrate experience-dependent plasticity and *elaborated integrative* functions. In our opinion, the presence of short and long-term plasticity features are essential features of neurons and, perhaps, many proneuronal cells.
(2) Neurons can make polarized and specialized connections (synapses) but do not necessarily do so in all their neurites and nervous circuits, as documented in basal metazoans and various bilaterians. Hormonal-like volume transmission in some cells (or neurites) can support many integrative functions without a specialized synapse and synaptic cleft. This happens if targets are localized within a few micrometers from transmitter release points or if fast chemical transmission is not required (e.g., for many small or sessile animals with limited motor reactions or for regulation of visceral/regenerative processes).
(3) Neurons are cells specialized for *integrating numerous signals* and information transmission functions. It was suggested that a neuron could express more genes, gene products, and especially ligand-gated receptors to support its integrative operations than other cell types (Moroz, 2009). Many homeostatic and signaling pathways in neurons can be redundant, and such expanded redundancy might also be a characteristic feature of neurons enabling greater plasticity and adaptability neural circuits. This is the easily testable hypothesis when one can directly quantify all genes expressed in given neurons (vs. other cell types) using next-generation sequencing technologies. Moreover, ensembles of neurons revealed novel emerging properties absent in other cell populations. Such emerging properties can form so-called neural syntax and endogenous self-maintaining oscillations and rhythms (Buzsaki, 2010; Hanson, 2021), which often separate ‘true’ neural systems from others.

Thus, we define neurons as a hierarchical ensemble of polarized heterogenous secretory cells *with synapses*, evolved for generation, integration, and directional propagation of electrochemical signals leading to the release of extracellular messengers—features that enable them to transmit information, primarily chemical in nature, beyond their immediate neighbors (at useful speed) and without affecting all intervening cells en route. Systemic decision-making, short- and long-term neuroplasticity as parts of learning and memory mechanisms are inherent components of any neural organization.

We do not know if all extant neurons have plasticity properties, but the development of phenotypic plasticity in terms of strength of synaptic transmission or (hyper) excitability might be an important trait for the evolution of nervous systems (Walters and Moroz, 2009). It would be intriguing to test whether ctenophores, placozoans, or sponges learn and remember. What kinds of non-associated and associated memory mechanisms exist in these lineages? In summary, not a single character, but a combination of features can be a better definition of neurons. Four components are critical to elaborate and clarify the used terms.

*First*, we emphasize the definition of neurotransmitters as signaling chemical messengers *not involved directly in nutrition* and related metabolic pathways (Miguel-Tome and Llinas, 2021). In such cases as glutamate and ATP, these presynaptically released molecules act on specific ligand-gated receptors in target cells. Only secondary, these molecules can contribute to cellular metabolism *via* uptake or transfer through other supportive/glia-type cells. This comment does not contradict the view that ancient usages of these molecules in cellular metabolism was the vital exaptation predating neuronal origins, which subsequentially led to neofunctionalizations and selection of such abundant metabolites as neurotransmitters (Moroz et al., 2021a; Moroz et al., 2021b).

*Second*, when metabolites ‘become’ signaling molecules, transmitters, and neurotransmitters, it tune natural selection processes toward their spatial distribution and novel functions in intracellular communications as rich information carriers. Indeed, in contrast to nutrients, *the receiver* (=postsynaptic cell) *does not ‘know’* what the signal value would be from the information standpoint (Miguel-Tome and Llinas, 2021). However, the selection of neurotransmitters includes chemical and past evolutionary history and bioenergetic constraints for preserving, eliminating, or expanding selected messengers in particular neural circuits and species (Moroz et al., 2021b; Moroz and Romanova, 2021).

*Third*, compartmentalizing hundreds of chemical communications enriches *the speed of decision-making* by neuronal ensembles (as a separate system with emerging properties) and communications to virtually *all* other systems within an organism. In other words, neurons extensively communicate *via* multiple receptors to ‘determine’ which signal to send or not to send to other cell types (Miguel-Tome and Llinas, 2021). Thus, the *elaborated integrative activity* (thousands of rapid communications between neurons) is the second hallmark of nervous systems, which separates them from other tissues and organs. In contrast to computers, the elaborated integrative activity of nervous systems is primarily based on the elaborated heterogeneity of its units (neurons) and a broad spectrum of chemically different intercellular messengers (rather than the usage of one excitatory and one inhibitory transmitters, for example).

The systemic exaptation leading to the origin of the neuronal organization was the ancestral recruitment of dozens and even hundreds of signal molecules in early animals. The *primordial transmitter diversity* scenario explains why even simpler extant nervous systems always have multiple neurotransmitters (rather than one or a few for depolarization or hyperpolarization responses in postsynaptic cells), as discussed elsewhere (Moroz et al., 2021b). Any nervous system comprises numerous neurotransmitter systems because neurons evolved from a broad diversity of functionally and genetically different secretory cells (Moroz, 2009; 2014a, 2021). Multiple transmitters and synapses physically and functionally ‘brought protoneurons together’. Transmitters and synapses ‘made and shaped’ nervous systems as we know them today in animals. The corollary of this hypothesis is the prediction that neural circuits in virtually unexplored ctenophores or integrative systems in nerveless animals such as placozoans composed of multiple polarized secretory cell types with dozens and even hundreds of transmitters. These predictions can be experimentally tested.

*Fourth*, *neurons evolved to learn and store information*, primarily by changing synaptic strength, wiring, and excitability. These features put neuroplasticity and memory mechanisms at the crossroad of animal adaptations supporting dynamic changes in stereotyped and learned behaviors to find new ecological niches and protection. The diversity of chemical transmission and synapses is an ideal playground to tune and further develop different forms of memory from the earliest stages of neuronal evolution. It is well-established that learning and memory mechanisms include biochemical and structural changes in synapses and excitability. There are multichemical cross-talks from pre- and postsynaptic cells using retrograde messengers (Kandel, 2001).

Furthermore, a complex dialog between synapses and the nucleus of a neuron leads to dramatic changes in gene expression programs and long-term (epigenetic) modification of the genome operation as the fundamentals of long-term memory (Kandel, 2001, 2009; Kandel et al., 2014; Asok et al., 2019). As a result, the combination of multi-transmitter and synaptic organizations, coupled with genome operation, provides the most exceptional communication, information transmission, and storage capabilities with the potential for countless emerging properties of neural systems in general. The rise of elementary intelligence and cognitive features are inherently coupled with synaptic wiring and evolved in parallel across many phyla.

Thus, we think the presence of chemical synapses is the most crucial feature of any extant neural/neuronal system. This criterion can separate canonical neuronal systems from other integrative systems in animals and beyond. For practical reasons, the criterion of the presence of chemical synapses taxonomically restricts the term neurons as an animal-specific innovation only. It contrasts and prevents confusions with different terminologies, like arguments for the existence of neural systems in plants and other eukaryotes [extensively reviewed in (Miguel-Tome and Llinas, 2021)]. We do not discuss artificial networks and systems, although we accept the hypothesis that cells with the function of neurons and synapse analogs can be discovered in other taxons (Miguel-Tome and Llinas, 2021) and potentially in other extraterrestrial life forms. See the appendix for some different definitions of neurons.

Again, on a practical note, the proposed incorporation of synapses in the definition of a neuron can also be a critical criterion that separates ‘true’ neurons/nervous systems from the so-called neuroid cells/neuroid systems in non-bilaterians (Figure 1, and Figure 2). Even considering the presence of syncytial organization within some neuronal elements in ctenophores (Sachkova et al., 2021), the past and recent electron microscopy reconstructions revealed the presence of numerous synapses with different secretory vesicles (Horridge and Mackay, 1964; Horridge, 1965; Hernandez-Nicaise, 1968; 1973b; a;c; 1974; Hernandez-Nicaise, 1991; Sachkova et al., 2021) reflecting the use of multiple transmitters.

The synapse-centered definition of neurons and nervous systems does not conflict with the presence of pure neurosecretory cells (without classical synapses like bag cells in the sea slug, *Aplysia* (Kupfermann, 1967, 1970) in nervous systems as the evolutionary conserved and the most ancient mode of integration. In earlier animals, (neuro) peptide/transmitter volume secretion was a remarkable proto-neuronal exaptation, and it is perfectly preserved and fully functional in all modern nervous systems (Moroz et al., 2021b). Many neurons have synaptic (highly localized) terminals and non-synaptic sites at different neurites, like in serotonergic metacerebral interneurons of Euthyneural gastropods (Weiss and Kupfermann, 1976; Gillette and Davis, 1977; Fuller et al., 1998; Sudlow et al., 1998), further stressing the importance of volume transmission for systemic integration of behaviors.

The participation of other cell types, including glia, in neuronal computations does not conflict with the definition of neurons proposed here. Indeed, vertebrate glial cells can communicate with each other through complex chemical signaling, cell adhesion molecules, and gap junctions (Bergles et al., 2000; Fields and Stevens-Graham, 2002; Bergles et al., 2010), but mammalian neurons and glia share the same path during neurogenesis, and neurons can be derived from glia (Noctor et al., 2001).

The origin of chemical synapses with a narrow synaptic cleft and adhesive highly localized molecules might be a dividing rubicon for the formation and rapid diversification of what we call canonical nervous systems today. We consider various secretory cells and volume transmission as the predecessor of neurons (Moroz, 2009, 2021). Still, all extant neural systems contain chemical synapses, at least in some parts, which is critical for more rapid, localized, and directional transmission. The evolution of synapses was based on combinatorics of the evolutionary conserved molecular modules (Ryan et al., 2008; Ryan and Grant, 2009; Arendt, 2020, 2021) involved in exocytosis and transmitter’s sensing with recruitments of diverse adhesive molecules evolved in unicellular and colonial eukaryotes for other functions (as exaptations). The presence of unique synapses and neurons in ctenophores suggests that the formation of the synaptic organization evolved more than once (Moroz and Kohn, 2016); see below.

Finally, the evolutionary making of the chemical synapse involved a dramatic reorganization of the endoplasmic reticulum, lipid diversifications, and compartmentalization within intracellular membranes, further promoting the rise of a neuronal organization (Moroz and Romanova, 2021). Molecular and functional classification of synapses, as performed in mammals (Emes et al., 2008; Grant, 2009; Masch et al., 2018; Roy et al., 2018; Zhu et al., 2018; Cizeron et al., 2020; Grant and Fransen, 2020; Bulovaite et al., 2022), is the most perspective direction to uncover the principles of neural ‘designs’ in basal metazoans.

## Electrical synapses in neural systems are less prominent compared to chemical transmission

Both canonical gap junction proteins and recently discovered tunneling nanotubes mediate a long-range junctional communication to coordinate metabolic coupling and signaling in a broad diversity of cells and tissues (Ariazi et al., 2017; Abudara et al., 2018; Guiza et al., 2018; Mayorquin et al., 2018). The electrical synapses or gap junctions also occur between neurons and might co-evolve with neurons (Ovsepian and Vesselkin, 2014; Ovsepian, 2017; Ovsepian et al., 2020). However, their fraction and contribution to the overall neuronal wiring are less prominent and often reflect no directional coupling. For example, in human brains, only about 1%–10% of connections are electrical (and mediated by connexins); the rest are chemical synapses. The same distribution is found in the model nematode (*C. elegans*), with about 10% of electrical synapses (mediated by innexins); the rest are chemical synapses.

Of note, invertebrate gap junctions were discovered first—since the name—innexins. Later, Panchin and others also discovered innexins in humans and other vertebrates (Panchin et al., 2000). Yuri Panchin proposed the new name, pannexins for this superfamily to unify both invertebrate and vertebrate innexins [from the Latin *pan*—all, throughout and *nexus* - connection, bond (Panchin et al., 2000)]. Although these two terms are synonymous, some distinct features of vertebrate pannexins (see below) lead to more often usage of this term for humans and mammals, while innexins continue to be broadly used for invertebrates.

Non-homologous classes of proteins (connexins and innexins/ = pannexins) make gap junctions (Baranova et al., 2004; Panchin, 2005; Abascal and Zardoya, 2013; Guiza et al., 2018) with the same membrane topology (Maeda et al., 2009; Oshima et al., 2016; Deng et al., 2020; Michalski et al., 2020; Qu et al., 2020; Ruan et al., 2020), reflecting their convergent evolution. Each hemichannel consists of six subunits for connexins and eight for innexins, and they are localized at opposite sides of two interacting cells (Oshima et al., 2016; Skerrett and Williams, 2017; Villanelo et al., 2017). Each hemichannel can be both homomeric (consisting of identical subunits) or heteromeric (different subunits), providing enormous combinatorial diversity of gap junctions: N^6^ for connexins and N^8^ for innexins (N = number of genes/isoforms).

In contrast to pannexins (Panchin, 2005), connexins were found only in tunicates and vertebrates (an apomorphy). The amphioxus genome encodes only one pannexin/innexin gene. This comparative distribution suggests that connexins evolved after early chordates lost innexins diversity (Welzel and Schuster, 2022). Three pannexins genes are present in mammals. Even so, they do not make electrical synapses because N-glycosylation in extracellular loops prevents interactions of hemichannels and the formation of functional junctions (Penuela et al., 2007; Sanchez-Pupo et al., 2018; Ruan et al., 2020; Welzel and Schuster, 2022). Pannexins hemichannels release various metabolites and signal molecules (such as ATP) from cell types (non-only neurons) with multiple functions (Panchin, 2005; Ransford et al., 2009; Scemes et al., 2009; D'Hondt et al., 2011; Sosinsky et al., 2011; Deng et al., 2020).

The best-studied system for the systemic neurobiology of innexins is the nematode *Caenorhabditis elegans*. According to its neuronal connectome reconstructions, 302 neurons can make 8693 synapses, but only 890 (10.2%) are electrical and formed by innexins. The *C. elegans* genome encodes 25 innexins, and 20 might contribute to neuronal wiring (Starich et al., 2001; Simonsen et al., 2014; Hall, 2017; Bhattacharya et al., 2019). The connectome of 282 somatic neurons contains 6393 interneuronal chemical synapses, 1410 neuromuscular chemical synapses, and 890 gap junctions (Varshney et al., 2011). Electrical synapses might have functional directionality and plasticity but at a limited scale. Thus, a reduced directionally of majority of studied electrical synapses might be one of the significant constraints, limiting their “expansions’ across neuronal populations within all phyla.

Quantitative analyses of two numerically similar networks in *C. elegans* further demonstrate relationships between electrical and chemical synapses in the nervous system. Chklovskii and others (Varshney et al., 2011) analyzed the gap junction network of 279 neurons that make 514 junction connections consisting of one or more junctions. This electrical circuit has about 2-fold more gap junctions than neurons, however, finding directionality and *heterogenous* postsynaptic targets was challenging. In contrast, the chemically wired network in the same species also consists of 279 neurons and 2194 directed connections implemented by one or more chemical synapses. This network contained about one order of magnitude extent of chemical synapses, which were directional, with more transmitters and receptors providing significantly more computational capability and communication flexibility. It might be why chemical synapses are the hallmark of the nervous system from the very beginning.

Complex directionality with localized wiring could be the features that enhanced speed and computational power in expanded neural circuits of larger animals vs. anticipated dominance of pure volume transmission in early/potentially smaller animals. 3D spatial information transmission in neural assemblies (Moroz et al., 2021b) is inherent for chemical synapses, even at potentially higher bioenergetic costs to the nervous system.

In addition to simpler forms of electrical coupling (de-/and hyperpolarization) mediated by gap junctions; electric fields can also mediate inhibitory synaptic action as in the Mauthner cell network (Faber and Pereda, 2018). However, chemical synapses execute significantly more complex excitatory and inhibitory actions, with unprecedented capability to amplify signals and recruit different signaling pathways. These features further increase information capabilities supporting more complex behavioral controls, learning and memory. Finally, chemical synapses provide greater redundancy and adaptability within neuronal circuits and systemic integration of visceral and somatic functions by adding functional and evolutionary robustness to neuronal architectures.

Admittedly, electrical and true chemical synapses co-evolved due to increased animal and behavioral complexities (Ovsepian, 2017; Ovsepian et al., 2020), with extraordinary phylum-specific diversification across the animal tree. During synaptogenesis, electrical synapses might be established first and promote the formation of chemical synapses in development (Ovsepian and Vesselkin, 2014).

Bioenergetic studies indicated that the nervous system is very costly. As a result, a preferential selection of some groups of transmitters vs. others might occur [i.e., favoring the preservation of neuropeptide signaling machinery and some low molecular weight transmitters such as glutamate (Moroz et al., 2021a; Moroz et al., 2021b)]. The adaptability of chemical synapses overcomes the higher bioenergetic cost of information processing.

## Origin and diversification of innexins

Innexins/pannexins-based junctions are not found in colonial and unicellular eukaryotes; therefore, they are metazoan but not neuron-specific synapomorphy. Phylogenomics survey pointed out that pannexins evolved in the common ancestor of all metazoans (Moroz et al., 2014) and intensely diversified in virtually every studied animal phylum, including ctenophores (Moroz and Kohn, 2016), cnidarians, and most protostome lineages (Moroz et al., 2014; Welzel and Schuster, 2022). These numerous events of *independent innexin radiation* correlate with the respective increases in tissue/organ differentiation and needs for physical/metabolic cell coupling *unrelated* to neuronal functions. The simpler bodyplans in placozoans and sponges are associated with the absence/loss of innexins and ‘needs’ of direct intercellular connections.

Gap junctions, recruited in the evolution of early animals, address the dramatic increase in the number of cell types (compared to colonial organisms), by coupling similar cell populations to mediate more stereotyped functions: secretion, contractility, coordinating cilia beating, exchange macromolecules and mRNAs during embryogenesis, regeneration, contribution to mechanistic tissue biology, *etc.* Thus, gap junctions are much more widespread across cell types and tissues (e.g., practically every cell in *C. elegans* expresses gap junctions) and broadly used to communicate between other cell types rather than neurons, including the integrative functions during development.

In ctenophores, innexins are very diverse and involved in numerous functions, from embryogenesis to behavioral control. Moroz and Kohn found that the genomes of both *Pleurobrachia bachei* and *Mnemiopsis leidyi* encode 12 innexins each (Moroz et al., 2014; Moroz and Kohn, 2016), potentially creating 429,981,696 combinations (12^8^). Analysis of ten ctenophore transcriptomes and two genomes showed that independent diversification of innexins occurred early in ctenophore evolution with several ctenophore-lineage-specific innovations (Moroz et al., 2014; Moroz and Kohn, 2016; Welzel and Schuster, 2022). This phylogeny suggests that ongoing adaptive radiation of gap junction proteins is associated with the profound diversification of the bodyplans within Ctenophora [pelagic vs. benthic species, active vs. passive predators, *etc.* (Whelan et al., 2017)]. Equally interesting is the finding that 67% of ctenophore innexins have N-glycosylation sites, potentially preventing the formation of gap junctions between cells as in chordates. These N-glycosylation sites also evolved independently in ctenophores because they are not conserved in all species within the phylum (Welzel and Schuster, 2022).

Moroz et al. (2014) compared the gene expression profiles of *Pleurobrachia* innexins during development and across all major adult tissues. Remarkable innexins’ expression was shown in early and later embryos and larvae of *Pleurobrachia* when the nervous system was not formed, but with the burst of expression for all 12 innexin genes in adults, including co-expression of several genes in the aboral organ, combs and tentacles (Moroz et al., 2014). Ctenophore gap junctions are likely responsible for communications in alternative conductive pathways, including combs (Satterlie and Case, 1978) and ciliated furrows (Figure 1), which G. A. Horridge originally called the neuroid cells. The aboral organ and distributed neural nets control and integrate alternative conducting pathways. Although jap junction-mediated interactions have not been studied in detail, the nervous system with chemical synapses occupies the top of a hierarchical organization of behaviors in ctenophores.

## Convergent evolution of synapses

Generalized extant neurons are cells that make chemical synapses among themselves and other cell types, with few exceptions. The first neural-like integrative systems were non-synaptic with volume transmission (Moroz, 2009; Moroz et al., 2021b; Jekely, 2021). Comparative data from nerveless animals and choanoflagellates support this hypothesis. All major components of presynaptic (secretory part) and postsynaptic (receptive part) modular machinery predate animals. (Sakarya et al., 2007; Kosik, 2009; Ryan and Grant, 2009; Conaco et al., 2012; Moroz and Kohn, 2015; Wong et al., 2019; Arendt, 2020; Ovsepian et al., 2020; Gohde et al., 2021). There are no pan-synaptic genes (Moroz and Kohn, 2015). A subset of evolutionarily conserved proteins is common to all neurons and synapses (exocytosis components, receptors, channels, transporters, *etc.*). Still, they are not unique neurons or synapses and are often co-opted for multiple functions. Genes encoding most of these proteins are broadly expressed across other non-neuronal cells and tissues; therefore, they have limited use as specific neuronal or synaptic markers, particularly in the analysis of non-bilaterian systems. This notion suggests: different adhesive molecules (e.g., cadherins, neuroligins, neurexins, *etc.*) and chaperons could subsequentially or in parallel scaffold individual protein modules to form chemical synapses with a defined synaptic cleft and its components.

The corollary of the neuronal polyphyly hypothesis is the scenario of independent origins of synapses (Moroz, 2009; 2014a). This scenario is supported by data about the distinct structural and presumed molecular organization of ctenophore synapses derived from genomic studies (Moroz and Kohn, 2016). However, the molecular composition of ctenophore synapses, their neurotransmitters, and signal molecules, in general, are largely unknown. The initial evidence exists for transmitter roles of glutamate (Moroz et al., 2014; Moroz and Kohn, 2015; Moroz et al., 2020b), nitric oxide (Moroz et al., 2020a), and ctenophore-specific neuropeptides in *Pleurobrachia*, *Mnemiopsis*, *Bolinopsis,* and kin (Moroz et al., 2014; Moroz and Kohn, 2015; Sachkova et al., 2021). Nevertheless, not a single synapse has been physiologically or molecularly characterized in ctenophores.

Earlier ultrastructural data revealed an unusual tripartite synaptic organization—the ‘presynaptic triad’ (Hernandez-Nicaise, 1973c, 1974; Hernandez-Nicaise, 1991). Each presynaptic region contains three layers of organelles: a layer of synaptic vesicles lining the presynaptic membrane, a cistern of agranular endoplasmic reticulum just above the row of vesicles, followed by one or several mitochondria. The non-polarized organization with the apparent ability to form synapses everywhere and symmetrical synapses was also demonstrated (Hernandez-Nicaise, 1973c; Hernandez-Nicaise, 1991) and confirmed by recent reconstruction (Sachkova et al., 2021; Burkhardt et al., 2022).

Synaptic systems in cnidarians and bilaterians are also quite different (Anderson, 1985; Anderson and Grunert, 1988; Anderson and Spencer, 1989; Anderson and Trapido-Rosenthal, 2009), with only partially overlapping subsets of neurotransmitters, receptors, and components of the synaptic cleft (Moroz and Kohn, 2015; Moroz et al., 2021b). Again, not a single synapse has been molecularly characterized in cnidarians, basal bilaterians, or any basal deuterostome. Thus, targeting synaptic systems in a taxonomically broad array of reference species would be both a discovery-driven enterprise and an opportunity to ask many intriguing questions, particularly about the directionality in information processing, learning and memory, and cellular bases of behavior across pre-bilaterians.

Considering more than one billion years of separate evolutionary paths for every phylum, we predict discoveries of fundamental differences across phyla regarding molecular diversity and the operation of synapses. Equally important would be the characterization of the volume transmission dynamic and its relationships with chemical synapses and other integrative systems in basal metazoans. The deciphering hierarchy within (neuro) transmitter systems and neural circuits is also a new direction of comparative research across all non-bilaterian lineages. It is an exciting time to unite multiple disciplines in such an endeavor.

In summary, little-explored examples of convergent neuronal evolution in early branching metazoans are essential to discover novel molecular and cellular toolkits controlling stereotyped and learned behaviors with prospects of neuro-engineering and synthetic biology. We also predict a greater diversity of neuroid and other systems in prebilaterian metazoans, including sponges and placozoans (Figure 2), than in Bilateria.

## Concluding remarks

We agree with the recent statement: “to advance our knowledge of the nervous system, we should adopt a physiological definition (Miguel-Tome and Llinas, 2021). From this perspective, we stress several significant points and testable hypotheses:

(1) Neurons (and nervous systems) are functional but not a genetic category.
(2) Any given neural system is not a single character; it includes different cell lineages with different genealogies and origins.
(3) Both centralized and distributed nervous systems could be chimeric and composed of highly heterogenous cell populations, perhaps with unrelated origins.
(4) There are no pan-neuronal/pan-synaptic genes, and synapses evolved more than once with different neurotransmitter systems and adhesion molecules forming the synaptic cleft.
(5) Neurons with different transmitters evolved independently from different secretory neuroid-like, digestive and/or immune-type cells that might already have these (or similar) transmitters (e.g., secretory peptides, GABA, monoamines, *etc.*) or components of relevant transmitter synthetic pathways. However, transmitter phenotype could be changed in development (Spitzer, 2017; Bertuzzi et al., 2018; Meng et al., 2018; Ferrarelli, 2020; Li et al., 2020) and evolution with co-option of peptide-type and low molecular weight neurotransmitters.
(6) Novel signaling molecules and neurotransmitter systems should exist in non-bilaterians, and their diversity might exceed the diversity of neurotransmitter systems in bilaterians. In other words, neural systems in early-branching metazoans are molecularly/genetically more heterogenous than more derived bilaterian neural ensembles, which could secondarily lose the primordial diversity of molecular players in the LCAA or the urbilaterian.
(7) Phylogeny of systemic memory is based on the early evolution of the synaptic organization; by characterizing neuroplasticity mechanisms in representatives of all four non-bilaterian lineages, it would be reconstructed. Some learning and memory mechanisms might be different in non-bilaterians expanding the scope of neuroplasticity.
(8) Degrees of emerging properties across nervous systems and neuronal ensembles significantly differ in basal metazoans, especially in ctenophores vs. bilaterians.
(9) Multiple alternative neuroid-like integrative systems are present in all early-branched metazoan lineages with distinct hierarchical organizations, contributing to these species’ learning and memory mechanisms.
(10) The role of neuroid-like integrative systems in cognition and behavior of bilaterians may be under-appreciated. (i.e., we may be overstating the role of neurons because of a misleading analogy between electronic computers and brains). (This comment was suggested by the reviewer, and we fully agree with this statement).

Considering minimal information about neural systems in ctenophores and alternative, integrative systems in sponges and placozoans, many fundamental questions about neuronal identity, plasticity and neuronal homology remained controversial. Based on expression data in sponges (Musser et al., 2021), their neuroid cells express a different subset of genes, which are distinct from other known neural cells in other animals. Thus, we implement a hypothesis of their independent origins and propose the terminology of alternative integrative/neural systems.

Given these proposed generalized features of neurons, it is reasonable to address the question: what is the molecular/genomic foundation that lets a cell be or not be a neuron?

The neural system evolved as a primary integrative system in organisms and considering the extreme diversity of microchemical microenvironments, the presence of multiple signal molecules, and numerous external signals; it would be reasonable to predict that most neurons are tuned to process multiple signals. Co-options of numerous (even redundant) signaling pathways might provide a versatile molecular playground for processing interneuronal communications and functions.

Evidence from some vertebrate and molluscan neurons, where deep single-cell sequencing were performed indicated the presence of several dozens of receptors within the same cell. However, as correctly indicated by one of the reviewers, “simpler” mechanoreceptive sensory neurons or specialized olfactory cells can be specialized to process a single signal. But such neurons do not exist alone, and they transmit information to interneurons and other cell types, which integrate numerous signals.

One of us earlier proposed that a complex and coordinated transcriptome/epigenomic response in a cell with co-expression of multiple genes (or even a majority of genes in a genome) at any given time is the primary requirement to be a neuron in the first place (Moroz, 2009). It might also be a significant component in developing the logic of gene regulation that drives neural evolution and the origin of various cell types in nervous and other integrative systems. What factors could initiate such a generalized, integrative, and adaptive transcriptome/epigenome response in earlier cells and promote the emergence of neuronal-like properties? The neurogenic role of injury in evolution can be one of these universal factors (Moroz, 2009; Moroz et al., 2021b). The memory of injury could be the earliest form of memory in evolution (Walters and Moroz, 2009). Similarly, defense/immune-type signaling involved in regenerative and morphogenic responses could be exaptations for early neurogenesis (Moroz, 2009; Fields et al., 2020; Moroz et al., 2021b). These and other hypotheses are testable by implementing comparative approaches with modern single-cell ‘omic’ technologies.

## Data Availability

The original contributions presented in the study are included in the article, further inquiries can be directed to the corresponding authors.

## Appendix: Selected examples of definitions of neurons and nervous systems

“By means of nerves, the pathways of the senses are distributed like the roots and fibers of a tree.” --Alessandro Benedetti, 1497. https://web.stanford.edu/class/history13/earlysciencelab/body/nervespages/nerves.html

Here, we provide diverse illustrations of particular neuronal features with some of our comments (all highlights are ours).

1) “nervous system, organized group of cells specialized for the conduction of electrochemical stimuli from sensory receptors through a network to the site at which a response occurs”—Britannica https://www.britannica.com/science/nervous-system - by Thomas L. Lentz, the author of *Primitive Nervous Systems* (Lentz, 1968)*.*

More expanded the same definition: ‘In animals, in addition to chemical regulation *via* the endocrine system, there is another integrative system called the nervous system. A nervous system can be defined as an organized group of cells, called neurons, specialized for the conduction of an impulse—an *excited* state—from a sensory receptor through a nerve network to an effector, the site at which the response occurs.’ https://www.britannica.com/science/nervous-system

2) “the bodily system that in vertebrates is made up of the brain and spinal cord, nerves, ganglia, and parts of the receptor organs and that receives and *interprets* stimuli and transmits impulses to the effector organs” https://www.merriam-webster.com/dictionary/nervous-system
3) “A nervous system might be defined as an organized constellation of cells (neurons) specialized for the *repeated* conduction of an *excited* state from receptor sites or from neurons to effectors or other neurons” (Bullock and Horridge, 1965).

- Inhibition and endogenous rhythmics are equally important information/signaling components.

4) “A nervous system might be defined as an organized constellation of nerve cells *and associated non-nervous cells*; it includes receptors, but not most effector cells. A corollary of the definition of nervous is that they differ both quantitatively and qualitatively from other organ systems, *because they deal only incidentally with material and energy*. Their function and specialization is to *process information*, and their organizational complexity *greatly exceeds* that of any other system.” (Bullock et al., 1977)

- Here, the critical note was added, which separates the usage of signal molecules for informational processing from the use of released other molecules for nutrition and bioenergetic purposes.

5) “Nerve cells—which we shell hereafter synonymously call neurons—may be defined as cells specialized for generation, integration, and conduction of *excited* states, including most sensory but not effector cells. A corollary of this definition of nerve cells is that they derive their excitation intrinsically or from the environment, from special sense cells, or from other neurons and deliver it to other excitable or to effectors such as muscle cells.” (Bullock et al., 1977).

- Inhibition and endogenous rhythmics are equally important information/signaling components.

6) “Neurons are heterogeneously shaped, highly active secretory cells.” (Squire et al., 2013)

- This definition stresses one of the essential features of neurons but is also applicable to some endocrine and other secretory [e.g., mucus or digestive-related secretion].

7) “Neurons are information processing devices that receive, integrate and transmit signals to induce specific patterns of behavior.” (Hobert, 2013)

- All of these functions can be applied to many cell types of nerveless animals. Even in nerveless animals such as *Trichoplax*, we can find cells that fulfill this definition of neurons (Figure 2). It would be necessary to stress that the remarkable chemical and secretory heterogeneity of cell types is the key to neural organization

8) The nervous system is defined by the presence of a special type of cell—the neuron (sometimes called “neurone” or “nerve cell”). Neurons can be distinguished from other cells in a number of ways, but their most fundamental property is that *they communicate with other cells via synapses*, which are membrane-to-membrane junctions containing molecular machinery that allows rapid transmission of signals, either electrical or chemical.—Wikipedia (https://en.wikipedia.org/wiki/Nervous_system#cite_note-KandelCh2-10) with reference to (Kandel et al., 2000) *“Ch. 2: Nerve cells and behavior”.*

9) Past and present views of neurons and research perspectives within frameworks of the Cajal Neuron Doctrine have been elegantly summarized in 2005 (Bullock et al., 2005). We provide three quotes relevant to the present discussion. “A neuron is an anatomically and functionally distinct cellular unit that arises through the differentiation of a precursor neuroblast cell. What has evolved is a modern view of the neuron that allows a more broad and intricate perspective of *how information is processed in the nervous system.*” “ …axon-glial communication violates the Neuron Doctrine in two ways. Information is communicated between cells at sites far removed from chemical synapses, and it propagates in a transduced form through cells that are not neurons”.

- The raised questions are fundamental to studying neural systems in invertebrates and basal metazoans, particularly where identifying glia-type cells is elusive. Equally, important would be the characterization of alternative integrative systems within the broad spectrum of organisms, as discussed in this manuscript.

10) ‘A nervous system is the system of a multicellular organism that 1) contains a group or groups of cells that are specialized in transmitting, generating or processing information, 2) sends signals to other systems, allowing the organism to react to or act upon exogenous and endogenous states by controlling those systems’ activity, and 3) generates and sends signals to other systems as the result of communication among multiple specialized cells of the system.‘ (Miguel-Tome and Llinas, 2021)

- This is the conceptually broadest definition of nervous system from a physiological standpoint rather than a phylogenetic perspective, as stressed by these authors (Miguel-Tome and Llinas, 2021). It incorporates plants and might become elusive since specific molecular and functional modules would be difficult to formalize. In any case, it stresses extensive parallel evolution and functional convergence of my integrative systems across all domains of life.

11) “Neurons as hierarchies of quantum reference frames” (Fieldsa et al., 2022)—This is an intriguing systemic definition of neurons to be further explored from physical and information viewpoints.
